# The accuracy of the report of hepatic steatosis on ultrasonography in patients infected with hepatitis C in a clinical setting: A retrospective observational study

**DOI:** 10.1186/1471-230X-5-14

**Published:** 2005-04-13

**Authors:** Matthew J Hepburn, Jeffrey A Vos, Eric P Fillman, Eric J Lawitz

**Affiliations:** 1 Departments of Medicine Brooke Army Medical Center, Fort Sam Houston Texas, USA; 2 Department of Pathology, Brooke Army Medical Center, Fort Sam Houston Texas, USA

## Abstract

**Background:**

Steatosis is occasionally reported during screening ultrasonography in patients with hepatitis C virus (HCV). We conducted a retrospective observational study to assess the factors associated with steatosis on ultrasonography and the relationship between steatosis on ultrasound versus biopsy in patients infected with HCV in a clinical setting. Our hypothesis was ultrasonography would perform poorly for the detection of steatosis outside of the context of a controlled study, primarily due to false-positive results caused by hepatic fibrosis and inflammation.

**Methods:**

A retrospective review of ultrasound reports was conducted on patients infected with HCV in a tertiary care gastroenterology clinic. Reports were reviewed for the specific documentation of the presence of steatosis. Baseline clinical and histologic parameters were recorded, and compared for patients with vs. without steatosis. Multiple logistic regression analysis was performed on these baseline variables. Liver biopsies were reviewed by two pathologists, and graded for steatosis. Steatosis on biopsy was compared to steatosis on ultrasound report, and the performance characteristics of ultrasonography were calculated, using biopsy as the gold standard.

**Results:**

Ultrasound reports were available on 164 patients. Patients with steatosis on ultrasound had a higher incidence of the following parameters compared to patients without steatosis: diabetes (12/49 [24%] vs. 7/115 [6%], p < 0.001), fibrosis stage >2 (15/48 [31%] vs. 16/110 [15%], p = 0.02), histologic grade >2 (19/48 [40%] vs. 17/103 [17%], p = 0.002), and ALT (129.5 ± 89.0 IU/L vs. 94.3 ± 87.0 IU/L, p = 0.01). Histologic grade was the only factor independently associated with steatosis with multivariate analysis. When compared to the histologic diagnosis of steatosis (n = 122), ultrasonography had a substantial number of false-positive and false-negative results. In patients with a normal ultrasound, 8/82 (10%) had >30% steatosis on biopsy. Among patients with steatosis reported on ultrasound, only 12/40 (30%) had >30% steatosis on biopsy review.

**Conclusion:**

Steatosis on ultrasound is associated with markers of inflammation and fibrosis in HCV-infected patients, but does not consistently correlate with steatosis on biopsy outside of the context of a controlled study. Clinicians should be skeptical of the definitive diagnosis of steatosis on hepatic ultrasonography.

## Background

Hepatic steatosis is commonly detected on both radiologic and histologic examination in patients infected with the hepatitis C virus (HCV). In particular, steatosis is often observed in genotype 3 infection [1]. The underlying mechanism of steatosis in hepatitis C is not completely understood, and is most likely multifactorial. Animal models [2,3] and *in vitro *experimentation [4] suggest that a virologic effect induces steatosis. Clinical observational data suggest that a possible direct virologic effect occurs with hepatitis C genotype 3 infection [1,5]. However, steatosis in genotype 1 and 2 HCV infections is more closely related to risk factors that are known to be associated with non-alcoholic steatohepatitis (i.e. diabetes mellitus and obesity)[5,6]. The assessment of steatosis may have clinical relevance, as some studies have suggested that the detection of steatosis can be an independent predictor of response to therapy [7].

Clinicians routinely order hepatic ultrasonography on patients with hepatitis C as an initial screening test for hepatocellular carcinoma or anatomic abnormality. In ordering this test, the radiologist may also detect and mention steatosis. However, the diagnosis of steatosis on ultrasound can be problematic, as the ultrasonic appearance of steatosis may be that of a 'bright liver,' which can also be observed with hepatic fibrosis and/or inflammation [8]. Even though these overlap syndromes exist, we observed a number of hepatic ultrasonography reports identifying steatosis in our clinical practice, as opposed to echogenic or 'bright liver'.

The sensitivity and specificity of ultrasonography for the detection of steatosis may be very high in the hands of an expert radiologist who consistently applies a particular criteria. However, many clinicians in the United States employ the services of a radiology department or group, in which many radiologists with varying levels of experience actually interpret the images. We were interested in the utility of a report of steatosis in this context.

Only one prior study has compared steatosis on ultrasound to histology in HCV-infected patients [9], and no correlation was observed between histologic and radiologic results. However, the conclusions of this study are limited since the diagnosis of HCV was based on the detection of HCV antibodies, rather than a determination of viremia with polymerase chain reaction (PCR) technology. Additionally, this study was not performed in the context of clinical practice.

We conducted a retrospective observational study to assess the radiology reports of hepatic ultrasonography of HCV-infected patients. Our purpose was to determine demographic, clinical and laboratory characteristics associated with radiology reports of steatosis on ultrasound in HCV-infected patients and to describe the association between steatosis on ultrasound and steatosis on liver biopsy. We specifically wanted to clarify the implications of the identification of steatosis on an ultrasound report for the practicing clinician. Our purpose was not to assess the performance characteristics of hepatic ultrasonography under controlled conditions. Rather, we were interested in the utility of these reports in routine clinical practice, with different radiologists over a three-year period. The first part of our analysis was to examine all patients who had hepatic ultrasonography performed, including patients who did not have a liver biopsy. The second part of our analysis (comparing steatosis on ultrasound report to steatosis on biopsy) included only patients who had hepatic ultrasonography and liver biopsy. We observed that ultrasound reports of hepatic steatosis were particularly associated with histologic inflammation, as well as fibrosis, and the sensitivity and specificity of steatosis on ultrasound was poor, when compared to steatosis on biopsy.

## Methods

### Patients

A retrospective review was performed of all patients who underwent screening hepatic ultrasonography seen in the hepatitis research clinic at our tertiary care, military academic medical center over a three-year period. Patients were those eligible for care at a military treatment facility, including active duty military personnel, their spouses, and military retirees (serving 20 or more years on active duty). All patients had hepatitis C infection, confirmed by serum HCV RNA PCR testing. Patients were referred from primary care clinics at our institution, and military primary care clinics throughout the region (Texas, Louisiana, Oklahoma, Kansas, Missouri and Colorado) for further assessment of their hepatitis C infection and for discussion of treatment options. The Institutional Review Board at Brooke Army Medical Center approved the study.

Demographic and Clinical Characteristics: Age, gender, body mass index (BMI) and ethnicity were recorded on each patient and utilized for analysis. The following laboratory parameters were noted: alanine aminotransferase (ALT), alpha-fetoprotein (AFP), and HCV genotype. Patients were considered to have diabetes if they were taking medication for diabetes treatment. Liver biopsy reports included a Metavir stage (0=no fibrosis to 4=cirrhosis) [10] and histologic grade (0–4, with 0=no inflammation to 4=severe inflammation), which were also included in the analysis. Each of these parameters was converted into a binomial variable, with 0–2 and 3–4 as the resultant two categories.

### Ultrasonography

All patients underwent hepatic ultrasonography during their initial evaluation if they did not have an ultrasound report from a prior assessment. Computerized records were reviewed for the ultrasonography reports. Ultrasonography was primarily performed at Brooke Army Medical Center, the same institution as our hepatitis research clinic. The machines utilized in the Radiology Department were the ATL Ultramark HDI 3000^® ^Ultrasound System and the ATL Ultramark HDI 5000^® ^Ultrasound System. A small number of patients in the hepatitis research clinic (<10%) are referred from Wilford Hall Air Force Medical Center, also located in San Antonio, Texas. If the patient had their ultrasound performed at the Air Force hospital, they were included in the analysis. The Radiology Departments at these two institutions (Brooke Army Medical Center and Wilford Hall Air Force Medical Center) maintain a combined accredited teaching program for radiology residents. These reports were scored as a binomial variable. If the ultrasound report mentioned steatosis as a finding, it was designated positive. If the ultrasound report did not mention steatosis, it was labeled negative. Equivocal studies containing phrases such as "possible steatosis" or "inflammation or steatosis" were excluded from the final analysis. Reports did not distinguish between diffuse or focal fatty liver.

### Histologic Examination

A liver biopsy was offered to patients during their initial evaluation in order to assess the patient's severity of disease. Liver biopsies were generally performed within 1–2 months of hepatic ultrasound. There were no liver biopsies in this study utilized that were > 6 months after ultrasonography. The initial histologic examination of the biopsy focused on the histologic stage (fibrosis) and grade (inflammation). For our study, two pathologists (J.A.V. and E.P.F.) retrospectively reviewed these biopsies and a percentage of steatosis was assigned to each biopsy specimen. Any steatosis was denoted as 1%, while a complete absence of steatosis was classified as 0%. If steatosis was considered >2%, it was recorded in 5% increments. These pathologists were blinded to ultrasound results, as well as any clinical characteristics of the patients. In order to explore the relationship between the report of steatosis on ultrasound compared to biopsy, the biopsies were categorized three different ways:

1) grouped into 5 categories (based on the percentage of steatosis on biopsy): 0–2%, 2–10%, 10–30%, 30–60%, and >60%.

2) As a binomial, comparing no steatosis (<2%) vs. any steatosis (>2%).

3) As a binomial, comparing significant steatosis (>30%) vs. not significant steatosis (<30%).

### Analysis

Descriptions of the 'operative characteristics' (sensitivity, specificity, positive predictive value, negative predictive value) of steatosis on ultrasound in our clinical setting were calculated, with the assumption that the gold standard for the diagnosis of hepatic steatosis was histologic examination. These calculations were performed for both the determination of any steatosis and significant steatosis on histologic examination. Any steatosis was defined as more than 2% steatosis on biopsy, while significant steatosis was defined as >30% steatosis on biopsy. Prior literature suggests that ultrasonography performs well in the diagnosis of significant steatosis [11].

Demographic, clinical and histologic characteristics were compared between patients with steatosis on biopsy versus patients without steatosis on biopsy. For continuous variables, an independent sample t-test was utilized if their distribution was normal. For continuous variables in which the distribution of values was not normal, the Mann-Whitney rank sum test was used. For categorical variables, a Pearson's chi-square test was employed. To determine the association between steatosis on ultrasound and steatosis on biopsy, a Spearman's rank correlation coefficient was calculated. A kappa-statistic was calculated to assess interobserver variability between steatosis percentages observed by the two pathologists. Agreement between pathologists was determined to be +/- 5% for the liver biopsies examined.

A multiple logistic regression model, with stepwise backward elimination of non-significant variables, was developed to determine factors that were associated with the detection of steatosis on ultrasound. Steatosis on ultrasound was the dependent variable, with the independent variables including: age, gender, histologic stage (as a binomial variable), histologic grade (as a binomial variable), ALT, genotype (genotype 3 compared to all other genotypes), body mass index, and a history of diabetes. All variables were included in the model.

## Results

Pre-treatment hepatic ultrasound reports were available on 171 patients. Seven reports were excluded because of equivocal descriptions (see Methods section, reports of "inflammation or fatty infiltration," or "steatosis vs. fibrosis" were excluded). A total of 49/164 (30%) of patients had steatosis documented in the ultrasound report. The comparison of baseline and clinical characteristics between patients with versus without steatosis on ultrasound indicated significant differences in ALT and AFP, a history of diabetes mellitus, and advanced histologic stage and grade (Table 1).

**Figure 1:**
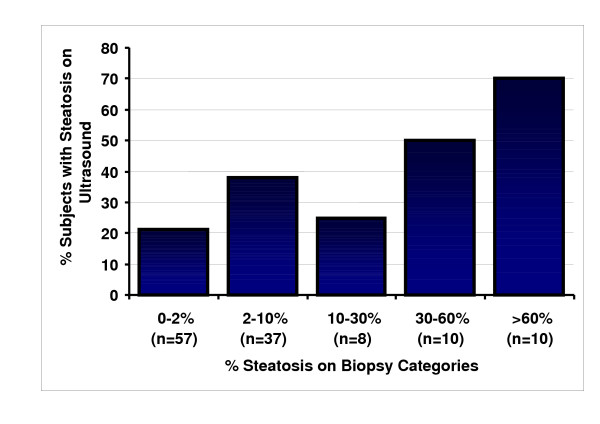
**Comparison of steatosis on biopsy versus ultrasound**. For each of the five categories of amount of steatosis on biopsy, the percentage of patients in those categories with a positive ultrasound for steatosis is displayed. r_s _= 0.27, p = 0.003.

Liver biopsy specimens were available from 122 patients for our pathologists to assess the amount of steatosis. The remaining 42 patients either did not have an initial liver biopsy (n = 6) or their liver biopsy slides were not available at our institution (n = 36), as they had been returned to the patient's referring institution. The kappa statistic for interobserver reliability of steatosis between the two pathologists was calculated to be 0.87. A relationship between steatosis on ultrasound and steatosis on biopsy was detected (Figure 1). The Spearman's rank correlation coefficient was r_s _= 0.27 (p = 0.003, n = 122). Of the 122 patients that had both histologic review and ultrasound, 40 (33%) had steatosis noted on their ultrasound report. A substantial number of false-positive and false-negative results were observed. For example, 10% (8/82) of patients with a normal ultrasound had >30% steatosis on biopsy. Among patients with steatosis reported on ultrasound, only 12/40 (30%) had >30% steatosis on biopsy review.

We determined the test characteristics for steatosis on ultrasound (in the clinical setting of our study) compared to biopsy as the gold standard, which were calculated for the detection of any steatosis (>2%), or for significant steatosis (>30%) (Table 2). For either of these calculations, the sensitivity and specificity of ultrasonography were not high. We also examined whether or not the diagnosis of steatosis on ultrasound was more useful in detecting substantial fibrosis (Metavir stage 3–4) on biopsy. The sensitivity of steatosis on ultrasound predicting fibrosis on biopsy was 48%, while the specificity was 74%. These performance characteristics for assessing fibrosis were very similar to the effectiveness of ultrasonography to correctly identify significant (>30%) steatosis on biopsy.

A total of 136 patients were included in the multivariate logistic regression model to determine factors associated with steatosis on ultrasound (see Methods for variables included in the model). The other 28 patients were not included due to a missing variable (genotype not performed, liver biopsy not performed, etc). The only statistically significant factor associated with steatosis on ultrasound was histologic grade (OR 3.6, 95% CI 1.3–9.8). A history of diabetes mellitus approached statistical significance (OR 3.8, 95% CI 0.96–14.70). No other factors were independently associated with steatosis on ultrasound.

## Discussion

The detection of steatosis on ultrasound in a clinical setting appears to be generally associated with steatosis on biopsy, but also with hepatic inflammation and fibrosis. In particular, steatosis on ultrasound was independently associated with moderate-severe histologic inflammation. The ability of ultrasonography to accurately detect hepatic steatosis is questionable outside of a controlled research setting, as both the sensitivity and specificity of this imaging technique were unacceptably low in our study. An ultrasound report of steatosis was as predictive of fibrosis as it was predictive of >30% steatosis, which reflects the unreliability of this imaging modality in differentiating fibrosis and steatosis. Based on the results of our study, the clinician should understand that an ultrasound report of steatosis could mean the patient has fibrosis, inflammation, significant steatosis, or a combination of these conditions. Alternatively, the patient may have none of these pathologic findings. Practitioners should rely on other diagnostic modalities to assess the liver for steatosis. Magnetic resonance imaging or computerized tomography are two potential techniques that may be clinically useful in the diagnosis of hepatic steatosis, pending further study [12].

Hepatic steatosis, detected by histologic examination, appears to have a multifactorial etiology in patients infected with hepatitis C. Steatosis may be a result of a direct virologic effect, particularly in patients infected with genotype 3 [1,5]. Additionally, steatosis in HCV-infected patients may be associated with accepted steatosis risk factors, including obesity [6,13,14], diabetes mellitus [6] and hypertriglyceridemia [13]. We observed that steatosis on ultrasonography was associated with factors representative of inflammation (histologic grade on biopsy and ALT) and fibrosis (histologic stage on biopsy and alpha-fetoprotein levels [15]) as well as diabetes mellitus. We did not observe an obvious association between genotype or body mass index with steatosis on ultrasound. Lack of association of genotype and BMI with steatosis in our study is likely related to factors confounding the diagnosis of steatosis on ultrasound, such as the additional presence of fibrosis and inflammation.

Increased echogenicity is the characteristic ultrasonographic finding that identifies hepatic steatosis. The increased echogenicity is often compared to the spleen and kidney [16,17]. A loss of definition of the hemi-diaphragm and decreased detail of the intrahepatic architecture (particularly the portal veins) may be supportive findings [11]. The performance characteristics of ultrasonography in the detection of steatosis vary considerably among studies. In the examination of patients with mostly alcoholic liver disease, a specificity of 84% and sensitivity of 94% for the detection of steatosis on ultrasound was described [18]. Using multiple criteria to diagnose steatosis, positive predictive values can be as high as 94% in high-prevalence populations [19]. Performance characteristics tend to improve with the diagnosis of moderate and severe steatosis [11]. One study suggested that 33% steatosis seen on biopsy was an optimal threshold for the radiographic detection of steatosis [20].

However, concomitant liver pathology may complicate the diagnosis of steatosis on ultrasound. Echogenicity on ultrasound may be consistent with either fibrosis or steatosis, and ultrasonography may not effectively differentiate between these two conditions [8]. Due to the overlap in appearance of fibrosis and steatosis, some radiologists opt to utilize the terms "fatty-fibrotic" [16,21] or "steatofibrosis" [22] when describing this echogenic pattern. Fibrosis has been demonstrated to be independently associated with steatosis in hepatitis C patients [6,14,23]. Some authors do not recommend the use of ultrasound as a screening tool for hepatic steatosis due to questions regarding sensitivity and specificity of this test [24].

Our results suggest that ultrasound is an unreliable predictor of steatosis when described on a routine ultrasound report in HCV-infected patients. These findings are consistent with a prior study of hepatic steatosis which documented no correlation between biopsy and ultrasonography in 64 patients with a positive HCV antibody [9]. Our results differed slightly from this previous report in that some correlation between ultrasound and biopsy was demonstrated (r_s _= 0.27). However, both false-positives and false-negatives were observed in our study. A report of steatosis was equally likely to indicate significant steatosis or fibrosis. Therefore, we conclude that hepatitis C produces significant liver pathology that may confound the diagnosis of steatosis on liver ultrasound in a clinical setting. It may be advisable for radiologists to report 'echogenic liver', 'possible steatosis vs. fibrosis' or 'bright liver', instead of definitively reporting the observation of steatosis.

For the clinician, ultrasound could conceivably be utilized in the documentation of an echogenic liver only. Similarly, any report of steatosis, fibrosis, or inflammation could be understood by the clinician as consistent with the presence of liver pathology. However, we also observed false negatives in our study, in which patients with significant steatosis had normal ultrasound reports. Additionally, the prognostic significance for clinical course and response to therapy may be very different for steatosis compared to fibrosis, and therefore the significance of an echogenic liver on ultrasound may vary substantially between patients.

Certain limitations of the study should be mentioned. The patients in this study had been referred for specialty evaluation in a hepatitis C clinic in which therapeutic clinical trials are emphasized. These patients may be different than the general population with HCV infection, limiting the generalizability of our results. Selection bias that is inherent in retrospective studies may limit the applicability of our results. Also, for some patients, there was time between the dates in which the ultrasound and biopsy were performed. It is possible that small changes in liver histology could have occurred, if the patient had significant weight gain or loss, for example. Ethanol consumption was not measured in our study, which could have impacted some of the baseline variables which were compared between patients with or without steatosis on ultrasound.

The retrospective nature of this study may introduce potential bias in data collection which could limit the clinical applicability of the findings. For example, some of the ultrasounds were not performed within the same 1–2 weeks as the liver biopsy. In addition, incorporating the radiologic interpretations of multiple radiologists (in contrast to a single radiologist) has certain advantages and disadvantages. While multiple radiologists potentially introduce significant variability in interpretation, they more accurately simulate a realistic clinical scenario as opposed to the artificial framework of a study utilizing a single expert radiologist. Since our study focused on the utility of ultrasonography results for clinicians, examining this question in the context of multiple radiologists over time seemed most appropriate. A similar review in other clinical systems, such as medical systems from other countries, would be useful. It is possible that in other systems, radiology do not attempt to define steatosis, but rather only remark on the presence and degree of an echogenic liver. It would also be interesting to survey radiologists to attempt to assess the range of techniques and criteria for the assessment of steatosis on ultrasonography. Additionally, liver biopsy itself is not always a definitive test, as sampling error can occur with this procedure. Differences in the assessment of histologic stage and grade have been observed in patients with hepatitis C who have undergone simultaneous liver biopsies in different lobes [25]. This error may account for some of the false-positive ultrasound reports in the assessment of steatosis in our study.

## Conclusion

Unfortunately, routine hepatic ultrasonography does not provide an accurate non-invasive mechanism for the diagnosis of hepatic steatosis in HCV-infected patients in the clinical context of our study. These findings should be examined in other clinical settings, perhaps in other countries. Clinicians should interpret a report of steatosis on ultrasound with caution, and also consider that this report could suggest a combination of inflammation, steatosis and/or fibrosis. Even patients who did not have a liver biopsy were included in the analysis of associations with steatosis, and the description of steatosis on ultrasound was associated with factors reflective of hepatic inflammation (such as ALT). Conversely, lack of ultrasonographic evidence of steatosis does not definitively exclude the presence of steatosis as shown on biopsy. Until a non-invasive modality is proven to be superior in a clinical setting, liver biopsy remains the optimal diagnostic procedure for the determination of steatosis in patients infected with hepatitis C.

## List of abbreviations

AFP Alpha-fetoprotein

ALT Alanine aminotransferase

BMI Body mass index

CI Confidence interval

HCV Hepatitis C virus

OR Odds ratio

PCR Polymerase chain reaction

RNA Ribonucleic acid

US Ultrasonography

## Competing interests

The author(s) declare that they have no competing interests.

## Authors' contributions

MH and EL developed the initial concept of the study. All authors participated in study design and data analysis. JV and EF conducted the histologic review of the slides. MH conducted the review of ultrasound reports and organization of the data. All authors contributed to the manuscript preparation and completion.

## Pre-publication history

The pre-publication history for this paper can be accessed here:



## Figures and Tables

**Table 1:** Comparison of demographic, clinical and laboratory characteristics of patients with versus without steatosis on hepatic ultrasound. Legend: ALT- alanine aminotransferase; AFP- alpha fetoprotein; BMI- body mass index.

**Parameter**	**Steatosis**	**No Steatosis**	**p value**
Gender (% male)	31/49 (63%)	77/115 (67%)	0.68
Genotype (3 vs. others)	4/46 (9%)	5/110 (5%)	0.31
Diabetes (% with DM)	12/49 (24%)	7/115 (6%)	0.001
Age	47.9 ± 10.3	46.3 ± 10.3	0.38
ALT (IU/L)	129.5 ± 89.0	94.3 ± 87.0	0.01
AFP (ng/ml)	13.1 ± 25.5	8.0 ± 12.3	0.03
BMI (kg/m^2^)	28.6 ± 5.1	27.4 ± 4.8	0.17
Stage (% stage 3–4)	15/48 (31%)	16/110 (15%)	0.02
Grade (% stage 3–4)*	19/48 (40%)	17/103 (17%)	0.002

*Seven histology reports scored only stage, and did not report a histologic grade.

**Table 2:** Performance characteristics of the detection of steatosis on ultrasound (n = 122). PPV = positive predictive value; NPV=negative predictive value. Gold standard was steatosis on histologic examination.

**Histologic Examination**	**Sensitivity**	**Specificity**	**PPV**	**NPV**
**Any Steatosis (>2%)**	43%	79%	70%	55%
**Steatosis >30%**	60%	73%	30%	90%
